# Correlation of cognitive functions of hypertensive patients on different classes of antihypertensive drugs

**DOI:** 10.5339/qmj.2025.106

**Published:** 2025-12-10

**Authors:** K. Deepalakshmi, S. Vijayabaskaran, K. Subhiksha

**Affiliations:** 1 Department of Physiology, PSG Institute of Medical Sciences and Research, Coimbatore, India *Email: drdeepalakshmik@gmail.com

**Keywords:** hypertension, cognition, angiotensin receptor blockers, calcium channel blockers, antihypertensive drugs

## Abstract

**Background:**

Globally, cognitive impairment has evolved as a major health and social issue. Hypertension, age, and stroke are independent risk factors for the development of cognitive impairment. Elderly individuals with hypertension are more prone to earlier cognitive decline. The risk is reduced by proper antihypertensive treatment. Various classes of antihypertensive drugs have varied protective effects on cognition, so we aimed to compare the cognitive scores of individuals taking different classes of antihypertensive drugs and correlate the blood pressure levels with their cognitive scores.

**Methods:**

Known hypertensive individuals belonging to the age group of 35 to 60 years participated in this single-center cross-sectional study conducted in a tertiary care center in southern India. Individuals with any chronic systemic illness or any history of pre-existing cognitive impairment were excluded from the study. After taking a detailed history on hypertension and drug history, the patient’s cognition was assessed using the Montreal Cognitive Assessment (MoCA) test. Out of a total score of 30, greater than or equal to 26 is considered normal. The hypertensive individuals were divided into three groups, namely those who were taking Angiotensin Receptor Blockers (ARBs), Calcium Channel Blockers (CCBs), and other antihypertensive drugs, and their cognitive scores were compared.

**Results:**

Two hundred and forty-nine known hypertensive individuals participated in the study, of whom 43% were found to be males. Patients on ARBs (25.1 ± 3) had higher cognitive scores compared to CCBs (22.6 ± 3.8) and other antihypertensive drugs (24.3 ± 3.8). When the cognitive scores were correlated with mean arterial pressure, it showed a negative association was shown (*P*-value, 0.45). When grouped individually and analyzed using an independent t-test, patients who have been hypertensive for more than 5 years and who were on irregular treatment showed lower cognitive scores, which is statistically significant.

**Conclusion:**

Our study showed a strong association between hypertension and cognitive function decline, and also the ARBs’ protective effect over other classes of antihypertensive drugs. The pharmacodynamics of different classes of antihypertensive drugs should be taken into account to support the cognitive health of hypertensive individuals.

## 1. INTRODUCTION

Globally, cognitive impairment has evolved as a major health and social issue as the life span of both men and women has increased dramatically in recent years.^1^ Cognitive skills occupy a vital role in an individual’s overall development and daily life, which includes thinking, reading, learning, retaining information, paying attention, solving problems, remembering tasks, and making decisions.

Cognitive impairment is also influenced by modifiable risk factors such as hypertension, diabetes mellitus, and hypercholesterolemia.^2^ The pooled prevalence of mild cognitive impairment (MCI) among hypertensive patients was found to be 30%, highlighting a substantial burden of cognitive impairment in this group. Subgroup analyses showed that prevalence rates varied by region, study design, age group, and diagnostic criteria. Notably, the prevalence was 26% in Asian populations and 40% in European populations.^3^ Cognitive disorders are classified along a spectrum based on severity, ranging from subtle changes to profound impairments as follows:^4^

- Subjective cognitive decline (SCD): Self-reported decline in memory or cognition without objective impairment on standardized testing.
- MCI: Measurable cognitive decline in one or more domains (e.g., memory, attention, executive function), greater than expected for age and education, but not severe enough to interfere significantly with daily activities.
- Dementia (major neurocognitive disorder): Significant decline in cognitive function that interferes with independence in everyday activities
- Delirium: Acute, fluctuating disturbance in attention and cognition.

Effects of hypertension on blood vessels accelerate arteriosclerosis, which decreases the blood flow to the brain tissue and brings about alterations in brain function, resulting in cognitive disabilities.^4,5^ Studies conducted among middle-aged hypertensive individuals reported increased risk of cognitive impairment by 20% to 54%, and a reduction in risk of dementia with appropriate antihypertensive treatment.^6^

MoCA—Montreal Cognitive Assessment is a validated screening tool used to detect MCI. It evaluates visuospatial skills, attention, language, abstract reasoning, delayed recall, executive function, and orientation.^7^ MoCA is a highly sensitive assessment tool utilized in clinical settings for early detection globally for academic and non-academic research.^8^ Mini-Mental State Examination (MMSE) and Addenbrooke’s Cognitive Examination (ACE-III) are also used for detecting MCI, but the Montreal Cognitive Assessment (MoCA) is considered to be more sensitive, particularly for identifying early cognitive changes

The drugs commonly used in the treatment of hypertension include angiotensin-converting enzyme (ACE) inhibitors, ACE receptor blockers, calcium channel blockers (CCBs), diuretics, vasodilators, beta blockers, and alpha blockers. Treatment with antihypertensives delays the cognitive impairment, but the degree of protective effect varies with different classes of antihypertensive drugs.^9^ Anti-hypertensives enhance the cognitive function by increasing the cerebral perfusion, by degrading the substance P, enkephalin, dynorphin, and neurotensin, reducing Aβ accumulation, and preventing calcium influx.^10^ It remains unclear which class of antihypertensive drugs is most effective and has the least side effects in preventing cognitive decline among hypertensive individuals. We aimed to compare the cognitive scores of individuals taking different classes of antihypertensive drugs and correlate the blood pressure levels with their cognitive scores.

## 2. METHODS

This cross-sectional study was conducted in a tertiary care center in southern India from October to December 2022. The study population was known hypertensive patients reported to the Internal Medicine outpatient department. The required sample size was calculated based on a correlation coefficient (*r*) of −0.15, with a significance level (α) of 0.05 and a power (1−β) of 0.80.

Study participants belonged to the age group of 35 to 60 years. Patients with hypertension for more than 2 years duration and on antihypertensive treatment with controlled and uncontrolled status were included in the study. Participants younger than 35 years of age and those suffering from co-morbidities such as diabetes mellitus, chronic respiratory diseases, chronic liver diseases, thyroid diseases, renal and neurological disorders, and hypertensive patients with a history of pre-existing cognitive impairment were excluded from the study. The study was initiated after getting approval from the institutional human ethical committee (PSG/IHEC/2022/Aprr/Exp/008) and informed consent from study participants. The study participants were subjected to the MoCA to test their cognitive function.

Detailed history such as family history of hypertension, duration of illness, smoking, alcohol intake (men who take more than two drinks per day and women who take one drink per day were defined as alcoholic according to the American Heart Association)^11^ diet, treatment history class of antihypertensive drugs was elicited and hypertensive individuals were divided into three groups according to class of antihypertensive drugs. Their cognitive scores were assessed by using the MoCA test.

### 2.1 Description of the cognitive tests

The MoCA^8^ is a rapid screening instrument to assess cognitive dysfunction. It assesses different cognitive domains: attention and concentration, executive functions, memory, language, visual-constructional skills, conceptual thinking, calculations, and orientation. The time duration to administer the MoCA is approximately 10 minutes. The MoCA test has a total score of 30. Study participants who scored ≥26 were classified as cognitively normal, while those with scores below 26 were considered to have cognitive dysfunction.

The domains assessed in the MoCA test were^12^:

- Visuo-constructional skills were assessed by asking the patients to draw an object (cube) within a given space. One point was allocated for a correctly executed drawing. The drawing must be three-dimensional, with all lines drawn accurately and without any additional or unnecessary lines. Lines should be relatively parallel and of similar length (rectangular prisms are acceptable). No points were awarded if any of these criteria were not met. The patients were asked to draw a clock and set the time. Scoring was done by assessing the following, which included contour—the clock must be in a circle with minimal imperfection. Numbers—all clock numbers must be present with no additional numbers and in the correct order. Hands—the hands must indicate the correct time; the hour hand must be shorter than the minute hand. The point was not assigned if any of the criteria were not satisfied.
- Naming function was assessed by asking the patients to name the animal (Three animals were shown, and each correct response carried one point).
- Memory was assessed by having the examiner read a list of five words at a rate of one word per second. The patients were then asked to recall the words, with a check mark given for each correctly recalled item. A second recall trial was conducted after a 5-minute delay (patients were asked to sit quietly). Scoring was based on the number of correctly recalled words across both trials.
- Attention was tested by asking the patients to repeat the numbers in forward and reverse order after giving the five random numbers and three random numbers, respectively. Scores were allotted for each correct sequence
- Vigilance was assessed by asking the patient to tap every time the examiner said letter A, when the examiner read the list of letters at a rate of one letter per second. The score was allotted accordingly
- Calculations were assessed by asking the patients to count by subtracting 7 from 100, and the patient was asked to keep on subtracting until the examiner asked the subject to stop. Five such numbers were elicited from each subject. Scoring was done according to the number of correct answers
- Sentence repetition was tested by asking the patients to repeat the sentence exactly, which was read by the examiner; 1 point was allocated for each sentence correctly repeated.
- Verbal fluency was tested by asking the patients to say as many words as possible that start with a particular alphabet letter in one minute. One point was given if the patients provided more than 11 words.
- Abstraction was assessed by asking the patients to explain what each pair of words had in common. Three such pairs of words should be tested; 1 point was allotted for each item pair correctly answered.
- Delayed Recall was tested by asking the patient to recall the words that he was asked to remember earlier. Scores were allotted for each word recalled freely without any cues.
- Orientation was assessed by asking the patients, details of the day, month, and year, and the place. Scoring was done according to the correct answers given by the patients

The sum of all scores was calculated.

## 3. RESULTS

A total of 260 patients were screened and invited to participate in the study. Seven patients did not give their informed consent, and four patients were found to have been diagnosed with hypertension for less than 2 years. Hence, 249 known hypertensive individuals who fulfilled the inclusion and exclusion criteria participated in this study, out of which 43% were males and 57% were females, with a mean age of 52.5 ± 6 and 53.3 ± 7.1 years, respectively.

The duration of illness was found to be more than 5 years for 88 (35%) patients. The treatment adherence history revealed, 209 (84%) patients were found to be compliant in taking antihypertensive medications (Table 1).

Hypertensive individuals were further grouped into three groups based on the class of antihypertensive drugs. The majority of the patients, 143 (57%), were receiving angiotensin receptor blockers (ARBs), 73 (29%) patients were on CCBs, and about 34 (14%) patients were taking other classes of drugs such as diuretics, ACE inhibitors, etc.

An independent t-test was employed to compare the cognitive scores of the patients based on the descriptive profile. Patients on ARBs (25.1 ± 3) had higher cognitive scores compared to CCBs (22.6 ± 3.8) and other antihypertensive drugs (24.3 ± 3.8). There was no statistically significant cognitive score difference between the patients’ groups based on sex, smoking history, alcohol intake, diet, and family history. Individuals who have been hypertensive for more than 5 years and who were on irregular treatment had lower cognitive scores, which were found to be statistically significant (Table 2). Higher cognitive scores were observed with individuals on ARBs when compared to individuals on CCBs (*P* < 0.001*) and other classes of drugs such as diuretics, ACE inhibitors, etc (*P* − 0.21). Lower cognitive scores were observed with individuals on CCBs when compared to individuals on other classes of drugs, such as diuretics, ACE inhibitors, etc., (*P* − 0.04; Table 2).

When the cognitive scores of hypertensive individuals were correlated with their mean arterial pressure using Pearson’s correlation test, there was a statistically significant negative correlation after adjusting for age (*P* value 0.04). The linear regression analysis (Pearson’s correlation) between the duration of the disease and their cognitive scores showed a statistically significant negative association among females. When the individual groups having specific traits were assessed for association between the duration of their disease and their cognitive scores, people who were on irregular treatment, people having a family history, and non-vegetarians exhibited a highly significant negative correlation (Pearson’s) after adjusting for age, gender, smoking, and alcoholism. People who are on regular treatment and people who don’t have any family history exhibited a modest negative correlation. Keeping cognitive scores as a binary variable (<26 categorized as having cognitive impairment), logistical regression was done, which also revealed a significant association (*P* < 0.001*) between the duration of the disease and the cognitive scores (Table 3). Their choice of anti-hypertensive therapy did not have any significant influence on this association (Table 4). Multi-collinearity test revealed no interactions between the assessed predictors (variant inflation factor < 10).

## 4. DISCUSSION

Hypertension results in both early vascular ageing and cognitive decline.^13^ Hypertension is an important risk factor for cognitive decline. Regular and appropriate intake of any class of antihypertensive drug that effectively reduces blood pressure was found to decrease the risk of cognitive decline, dementia, and Alzheimer’s disease (AD) in patients with hypertension.^14^

In our study, we observed a statistically significant negative correlation between the mean arterial pressure and cognitive scores of the hypertensive individuals. It was observed in some previous studies that there was a strong association between high blood pressure and a reduction in cognitive function.^15–17^ Several epidemiologic studies have also stated a stronger association as well as an inverse relationship between hypertension and cognitive decline.^18,19^ We also observed a significant negative correlation between their mean arterial pressures and cognitive scores in individuals who have been hypertensive for more than 5 years.

Our study results also revealed that individuals who have been hypertensive for more than 5 years and on irregular treatment had lower cognitive scores. A study conducted by Starr et al concluded that adequate blood pressure control reverses cognitive impairment.^20^ In a systematic review, authors had opined that antihypertensive treatment had some beneficial effects on cognitive decline and prevention of dementia, and also these effects vary between different classes of antihypertensive drugs, and ARBs showed the most beneficial effect.^21^

In our present study, we found that patients on CCBs had the lowest cognitive scores when compared with people on ARBs and other classes of drugs, such as diuretics and ACE inhibitors. Our study results were in line with a population-based cohort study done among 24,531 matching pairs of ARB and non-ARB exposed people conducted by Chiu et al., which demonstrated that patients on ARBs had more protection from dementia and cognitive defects.^22^ In another study conducted among the Japanese American elderly population, it was found that usage of beta blockers had a more beneficial effect on cognitive function. The use of β-blockers as the only antihypertensive medication at baseline was consistently linked to a reduced risk of cognitive impairment with an incidence rate ratio of 0.69; 95% confidence interval [CI] 0.50–0.94, compared to men not receiving any antihypertensive treatment, after adjusting for multiple potential confounding factors.^23^ Results of various studies underscore the importance of implementing comprehensive strategies that combine effective hypertension management with routine cognitive assessments to support healthy aging and maintain cognitive function.^24^

### 4.1 Limitations

The cross-sectional design of this study limits the ability to establish a cause-and-effect relationship between hypertension and cognitive impairment. Longitudinal studies are needed to evaluate temporal associations and track the progression of cognitive decline over time. Additionally, as the data were collected from a single center, the findings may not be generalizable to the broader population. The absence of follow-up further restricts insights into whether the observed cognitive deficits progress to dementia or improve with interventions. Control over potential confounding variables such as educational background, socioeconomic status, depression, sleep disturbances, and comorbidities like diabetes was limited, which may have influenced the outcomes. Moreover, self-reported information on medication adherence and cognitive complaints may be subject to recall and reporting bias.

### 4.2 Conclusion

Our study found a strong association between hypertension and decline in cognitive function, and ARBs had a protective effect over other classes of anti-hypertensives. Understanding the patterns of cognitive dysfunction in hypertensive individuals, along with the impact of different classes of antihypertensive drugs on cognitive function, can aid in preventing or delaying cognitive decline and ultimately enhance the quality of life for these patients.

## AUTHORS’ CONTRIBUTION

KD: Conception and design of the study, Analysis and interpretation of data, Drafting the article, Critical revising, Final approval. SV: Conception and design of the study, Acquisition of data, Analysis and interpretation of data, Drafting the article, Critical revising, Final approval. KS: Acquisition of data, Analysis and interpretation of data, Critical revising, Final approval.

## ETHICAL APPROVAL

Research was conducted in accordance with the ethical standards of the responsible committee on human experimentation (institutional and national) and according to the WMA declaration of Helsinki – ethical principles for medical research involving human subjects (ethical approval no.: 22/008 dated 31.1.2022).

## INFORMED CONSENT

Informed obtained from study participants based on the WMA declaration of Helsinki – ethical principles for medical research involving human subjects.

## CONFLICT OF INTEREST

The authors have no conflicts of interest to declare.

## DATA AVAILABILITY STATEMENT

Data sharing is not applicable.

## Figures and Tables

**Table 1.** Descriptive profile of the hypertensive study population.

Groups	Subgroups	*N*	Percentage
Sex (age)	Male: 52.5 ± 6 years	108	43%
	Female: 53.3 ± 7.1 years	141	57%
Treatment duration	<5 years	161	65%
	>5 years	88	35%
Treatment adherence	Regular	209	84%
	Irregular	40	16%
Anti-hypertensive drugs	Angiotensin receptor blockers	143	57%
	Calcium channel blockers	72	29%
	Others	34	14%
Smoking history	Yes	32	13%
	No	217	87%
Alcohol intake*	Yes	34	14%
	No	215	86%
Diet	Non vegetarians	202	81%
	vegetarians	47	19%
Family history of hypertension	Yes	68	27%
	No	181	73%

*Men who take more than two drinks per day and women who take more than one drink per day.

**Table 2.** Comparison of cognitive scores in the hypertensive study population based on descriptive profile.

Groups	Subgroups	*N*	Mean ± standard deviation	*P* value
Sex	Male	108	24.4 ± 3	0.65
	Female	141	24.1 ± 3.8	
Treatment duration	<5 years	161	24.9 ± 3.2	<0.001*
	>5 years	88	23.1 ± 3.7	
Treatment adherence	Regular	209	24.6 ± 3.3	<0.001*
	Irregular	40	22.1 ± 3.7	
Anti-hypertensive drugs	Angiotensin receptor blockers	143	25.1 ± 3	<0.001*
	Calcium channel blockers	72	22.6 ± 3.8	
	Angiotensin receptor blockers	143	25.1 ± 3	0.21
	Others	34	24.3 ± 3.8	
	Calcium channel blockers	72	22.6 ± 3.8	0.04*
	Others	34	24.3 ± 3.8	
Smoking history	Yes	32	24.8 ± 2.8	0.35
	No	217	24.2 ± 3.6	
Alcohol intake*	Yes	34	24.7 ± 3	0.43
	No	215	24.2 ± 3.6	
Diet	Non vegetarians	202	24.2 ± 3.5	0.79
	Vegetarians	47	24.4 ± 3.6	
Family history of hypertension	Yes	68	24.15 ± 3.3	0.8
	No	181	24.3 ± 3.6	

*Men who take more than two drinks per day and women who take more than one drink per day.

**Table 3.** Correlation between duration of hypertension and the cognitive scores.

Subjects		*N*	*r* value	*P* value
All participants		249	0.32	<0.001*
Males		109	0.24	0.21
Females		141	0.37	<0.001*
Adherence	Regular	209	−0.19	0.02*
	Irregular	40	−0.45	0.006*
Diet	Vegetarians	47	0.22	0.01*
	Non Vegetarians	202	−0.42	<0.001*
Family history	Absent	181	−0.28	0.01*
	Present	68	0.43	0.008*

*P < 0.05 is significant after adjusting for age, gender, adherence, smoking, alcohol intake, diet, and family history.

**Table 4.** Correlation between cognitive scores of the patients taking different classes of antihypertensive drugs.

Patients on	*n*	*r* value	*P* value
Angiotensin receptor blockers	143	0.08	0.23
Calcium channel blockers	72	0.31	0.08
Other drugs	34	0.32	0.21

*P < 0.05 is significant after adjusting for age, gender, smoking, and alcohol intake.
